# Supplementary material for: Interactions with Nature, Good for the Mind and Body: A Narrative Review
Source: Int J Environ Res Public Health. 2024 Mar 12;21(3):329. doi: 10.3390/ijerph21030329 (PMC10970260; doi:10.3390/ijerph21030329)
Supplement: Supplementary file 1 [file ijerph-21-00329-s001.zip › ijerph-2875094-supplementary.pdf]

Supplemental Table S1. Overview of Identified Studies

	Ref.	Author(s) (Year)	Population	N	Location	Study type	Health Outcome or Behavior	Intervention description (if applicable)	Results
Indirect Interactions									
	13	Li et al (2021)	Incarcerated men	326	China	Cross-sectional	Mental Health		Having a view of nature positively associated with distress tolerance, life satisfaction, and wellbeing and inversely associated with depression, anxiety, and loneliness.
	14	Michels et al (2022)	18 - 30-year-old adults	92	Belgium	Intervention	Mental Health and Dietary Behavior	Participants were exposed to one of four conditions: pictures of grayscale objects, pictures of green objects, pictures of grayscale plants, or pictures of green plants. Food choices, stress, affect, and food cravings measured before and after.	Greater increase in positive affect when looking at pictures of nature. Looking at plants associated with increase in desire to consume fruits and decrease in wanting to consume and preference for snacks.
	15	Chan et al (2021)	Undergraduate students, senior adults	46	Singapore	Intervention	Mental and Physical Health	Participants "walked" in nature or urban environment via virtual reality for 3-5 minutes	"Walking" in nature via virtual reality is positively associated with positive affect and inversely associated with negative affect. Heart rate decreased after "walking" in nature.

Ref.	Author(s) (Year)	Population	N	Location	Study type	Health Outcome or Behavior	Intervention description (if applicable)	Results
16	McAllister et al (2017)	Adults	220	Australia	Intervention	Mental Health	Participants watched a video of wild nature, urban nature, or non-nature control for about 2 min, 30 seconds	Significantly greater positive affect and perceived restorativeness among individuals who viewed wild nature videos compared to urban nature or control videos.
17	Saadatmand et al (2013)	Adults under mechanical ventilator support	60	Tehran, Iran	Intervention	Mental and Physical Health	Participants enrolled in the intervention (n=30) listened to pleasant nature sounds for 90 minutes. Control group (n=30) wore headphones and sat in silence for 90 minutes.	Lower systolic and diastolic blood pressure, anxiety, and agitation in intervention group.
18	Nguyen and Brymer (2018)	Adults	48	Australia ; Southeast Asia	Intervention	Mental Health	Two, 10-minute guided imagery conditions: nature or urban environment. Participants experienced each condition.	Greater reduction in anxiety after nature guided imagery.
19	Tabatabaie et al (2019)	Adults	469	Colorado , United States	Cross-sectional	Physical Activity		Positive relationship between perceived amount of greenspace and engagement in moderate to vigorous physical activity.
28	Gilchrist et al (2015)	Employees	366	United Kingdom	Cross-sectional	Mental Health		Being satisfied with view of nature positively associated with mental wellbeing.
29	Lottrup et al (2013)	Employees	402	Denmark	Cross-sectional	Mental Health		Being satisfied with view of nature associated with 2.13 greater odds for work capacity and 3.03 greater odds of job satisfaction.

Ref.	Author(s) (Year)	Population	N	Location	Study type	Health Outcome or Behavior	Intervention description (if applicable)	Results
30	Bodin et al (2015)	Adults	2612	Sweden	Cross-sectional	Mental Health and Sleep		Having windows that face out to nature associated with greater concentration. Having window(s) in the bedroom that face out to nature associated with lower risk of poor sleep quality.
32	Ziabari et al (2023)	Health Care Providers	406	Gilan, Iran	Cross-sectional	Mental Health		Having a view of nature inversely associated with emotional exhaustion, depersonalization, reduced personal accomplishment, and burnout.
33	Soga et al (2021)	Adults	3000	Tokyo, Japan	Cross-sectional	Mental Health		Having a view of nature positively associated with life satisfaction, self-esteem, and happiness, and inversely associated with depression, anxiety, and loneliness.
34	Bi et al (2022)	Adults	508	Xi'an, China	Cross-sectional	Mental Health		Having a view of nature positively associated with life satisfaction, and inversely associated with depression and anxiety.
36	Braçe et al (2020)	Adults	479	Carmona, Spain	Cross-sectional	Mental Health		Not having a view of nature positively associated with experiencing depression.
37	Pouso et al (2021)	Adults	6080	Worldwide	Cross-sectional	Mental Health		Not having a view of nature associated with greater odds of depression.
39	Dzhambov, Markevych,	Students	720	Bulgaria	Cross-Sectional	Mental Health		Perceived greenspace associated with better mental health.

Ref.	Author(s) (Year)	Population	N	Location	Study type	Health Outcome or Behavior	Intervention description (if applicable)	Results
	Hartig et al (2018)							
40	Han and Hyun (2019)	Adults	604	South Korea	Cross-sectional	Mental Health		Perception of indoor and outdoor nature associated with mental health and emotional wellbeing.
41	Rüger et al (2023)	Adults	1219	Germany	Cross-sectional	Mental Health		Well-being associated with quality of and access to nature.
42	Hipp et al (2016)	Students	439	United States and Scotland, United Kingdom	Cross-sectional	Mental Health		Perceived greenness on campus associated with quality of life.
43	Reid et al (2022)	Adults	903	Colorado, United States	Cross-sectional	Mental Health		Perceived amount of greenspace inversely associated with stress, depression and anxiety.
44	Feng and Astell-Berg (2018)	Women	3897	Australia	Longitudinal	Mental Health		Perception of greenspace associated with fewer symptoms of psychological distress and odds of serious mental illness.
45	Pope et al (2018)	Adults	578	Sandwell, United Kingdom	Cross-sectional	Mental Health		Decreased risk of psychological distress associated with perceived accessible greenspaces, the ability to relax in greenspaces, and sufficient greenspaces.
46	Cleary et al (2019)	Adults	5014	Brisbane, Canada	Longitudinal	Mental Health		Perceptions of green space was a significant predictor of psychological wellbeing.
47	Putrik et al (2015)	Adults	9879	Maastricht,	Cross-sectional	Mental and		Satisfaction with greenspace associated with mental and general health.

Ref.	Author(s) (Year)	Population	N	Location	Study type	Health Outcome or Behavior	Intervention description (if applicable)	Results
				Netherla nds		Physical Health		
48	Stangierska et al (2022)	Adults	381	Poland	Cross-sectional	Mental Health		Satisfaction with greenspace associated with quality of life.
49	Yeh et al (2017)	Adults	30	England, United Kingdom	Intervention	Mental Health	Participants ran on a treadmill and viewed a video of a nature scene, a picture of a nature scene or took part in their own entertainment.	Participants that took viewed the nature video and picture reported greater happiness after the intervention than the those the self-selected their entertainment.
50	Chang et al (2021)	Adults	44	Hong Kong, China	Intervention	Mental Health	Participants looked at pictures of an urban greenspace.	Viewing urban green landscapes associated with regions of the brain that is implicated with executive attention.
51	Hüfner et al (2020)	Adults	498	Austria	Intervention	Mental Health	Participants viewed an alpine or neutral environment.	Significant increase in emotional response when viewing alpine environment.
53	Sun et al (2023)	Pregnant women	63	Beijing, China	Intervention	Mental and Physical Health	Participants were immersed in one of three environments (non-green, moderately green, and highly green) for 5 minutes using virtual reality.	Significant increase in positive affect, and significant decrease in negative affect, systolic and diastolic blood pressure, skin conductance, heart rate, cortisol, and alpha amylase after experiencing green space environments.

Ref.	Author(s) (Year)	Population	N	Location	Study type	Health Outcome or Behavior	Intervention description (if applicable)	Results
54	Anderson et al (2023)	Adults	43	Hawaii, United States	Intervention	Mental Health	Participants viewed virtual reality nature scenes for 15-20 minutes, multiple times during their six month deployment.	No significant changes in positive or negative affect.
57	Catissi et al (2023)	Chemotherapy patients	173	Brazil	Intervention	Mental Health	Intervention group watched a 12-minute nature video. Control group did not watch any video.	Both groups reported decreased negative affect after the intervention. Intervention group reported improvement in pain, tiredness, depression, anxiety and appetite after the intervention.
58	Cadogan et al (2023)	Students	147	Cork, Ireland	Intervention	Mental Health	Participants watched a video of nature or urban environment.	Significant decrease in negative affect and rumination for those that watched the nature videos.
59	Hu et al (2022)	Front-line workers	71	Zhejiang, China	Intervention	Mental Health	Participants watched a video of a nature or urban scene for two minutes for five days.	Significant increase in positive affect and satisfaction with life among those that viewed the nature videos.
60	Kinnafick and Thøgersen-Ntoumani (2014)	Adults	40	United Kingdom	Intervention	Mental Health	Participants walked or sat while viewing a nature or urban scene.	Viewing a nature scene, regardless of the activity being performed, reduced negative affect.
61	Aghaie et al (2014)	Adults	120	Tehran, Iran	Intervention	Physical Health	Participants in the intervention group listened to nature-based sound therapy while being weaned off mechanical ventilation.	Significant lower levels of agitation and anxiety in the intervention group after being weaned off mechanical ventilation.
62	Aktas et al (2018)	Women	120	Ankara, Turkey	Intervention	Mental Health	Participants in intervention group listened to nature	Significant decrease in anxiety after gynecological exam for

Ref.	Author(s) (Year)	Population	N	Location	Study type	Health Outcome or Behavior	Intervention description (if applicable)	Results
							sounds while undergoing gynecological exam.	those that listened to nature sounds.
63	Kiper et al (2022)	Post-stroke survivors	60	Poland	Intervention	Mental Health	Virtual reality group received 10 sessions of 20 minute virtual reality therapy. Control group received 10 sessions of 20 minute Schultz's Autogenic Training.	Participants who received virtual reality therapy had significantly greater reduction in depressive symptoms.
64	Ginns et al (2023)	Students	72	Australia	Intervention	Mental Health	Participants had an unstructured break, watched a video, or did not have a break.	No significant difference between watching a video of nature and taking an unstructured break on directed attention or problem-solving.
65	Elsadek et al (2020)	Adults	30	Shanghai, China	Intervention	Physical Health	Participants looked out of building into urban and natural space for 3 minutes	Greater parasympathetic activity dominance and heart rate variability and lower skin conductance when looking at nature.
66	McSweeney et al (2021)	Undergraduate students	147	Canada	Intervention	Physical Health	Participants sat in a room with a view of urban-nature view, plants, pictures of nature, and diffuser or in a room without these elements (control) for 45 minutes.	Greater parasympathetic activity dominance and decreased heart rate among participants who had the nature condition.

Ref.	Author(s) (Year)	Population	N	Location	Study type	Health Outcome or Behavior	Intervention description (if applicable)	Results
67	Wang et al (2019)	Students and social workers	96	China	Intervention	Physical Health	After performing a stress test, participants viewed one of seven nature scenes for 5 minutes, using virtual reality.	Significant decrease in systolic and diastolic blood pressure after viewing nature. Observed significant increase in alpha amylase in one condition.
68	Hsieh et al (2023)	Students	45	Taiwan	Intervention	Physical Health	Participants were immersed in virtual reality and listened to high- or low- decibel nature sounds or no noise.	Listening to high-decibel nature sounds increase parasympathetic nervous activity. Heart rate decreased while being immersed in nature.
69	Benz et al (2022)	Students	150	Konstanz , Germany	Intervention	Physical Health	Participants that were part of the intervention watched a 10-minute nature video.	Increased heart rate variability and deceased heart rate after watching nature videos.
70	Encho et al (2023)	Older Adults	204	Japan	Cross-sectional	Physical Activity		Perception of greenspace associated with more time walking.
71	Sugiyama et al (2013)	Adults	681	Australia	Longitudinal	Physical Health		Perceptions of the presence and proximity to green spaces were significantly associated with a higher likelihood of walking maintenance.
72	Soares et al (2020)	Adults	247,54 8	Brazil	Cross-sectional	Physical Activity		Perception of greenspace associated with weekly

Ref.	Author(s) (Year)	Population	N	Location	Study type	Health Outcome or Behavior	Intervention description (if applicable)	Results
								frequency and daily duration of physical activity.
73	Ali et al (2017)	Adults	20,765	13 countries	Cross-sectional	Physical Activity		Perception of greenspace not significantly with physical activity.
74	Van Cauwenberg et al (2015)	Mid-Older Adults	2700	Australia	Longitudinal	Physical Activity		Subjective park proximity associated with walking.
75	Ward et al (2020)	Older Adults	10,540	Ireland	Cross-sectional	Physical Activity		Difficulty getting to green area associated with lower engagement in physical activity.
76	Zhang et al (2023)	Students	76	China	Intervention	Mental Health	Participants cycled indoors while looking at a tree, green or white wall.	Significant decrease in total mood disturbance when viewing a tree.
77	Jones and Wheat (2023)	Adults	12	United Kingdom	Intervention	Mental Health	Participant exercised with no stimuli, with a 360 degree video of nature, or being immersed in virtual reality.	Individuals who exercised in the 360 degree video expressed greater pleasure.
78	Mavros et al (2022)	Adults	42	Singapor e	Intervention	Mental Health	Participants walked on a treadmill and viewed videos of indoor, outdoor, and green environments.	Green space perceived as calm and positive.
80	Baena-Extremera et al (2021)	Adults	49	Spain	Intervention	Mental Health	Outdoor and indoor exercisers viewed pictures of pictures of nature and urban areas.	Outdoor exercise group exhibited greater attention and lower stress than indoor exercisers.

	Ref.	Author(s) (Year)	Population	N	Location	Study type	Health Outcome or Behavior	Intervention description (if applicable)	Results
Incidenta	20	Dzhambov et al (2021)	Students	323	Plovdiv, Bulgaria	Cross-sectional	Mental Health		Having houseplants positively associated with fewer symptoms of depression and anxiety.

Ref.	Author(s) (Year)	Population	N	Location	Study type	Health Outcome or Behavior	Intervention description (if applicable)	Results
82	Maury-Mora et al (2022)	Adults	132	Madrid, Spain	Cross-sectional	Mental Health		Individuals who had indoor plants experienced less stress, compared to those who had access to greenspaces.
83	Ma (2022)	Adults	421	China	Cross-sectional	Mental Health		Mental wellbeing and mindfulness positively associated with hours of caring for houseplants, the number of houseplants in the home, and years of caring for houseplants
84	Pérez-Urrestarazu et al (2021)	Adults	4205	Brazil, Greece, Spain, Italy	Cross-sectional	Mental Health		Having more houseplants has been reported with greater emotional wellbeing.
85	Lee et al (2015)	Adult men	24	South Korea	Intervention	Mental and Physical Health	Participants worked on a document on a computer and transplanted a plant.	After transplanting a houseplant participants reported greater feelings of relaxation and comfort and had significantly less sympathetic activity and diastolic blood pressure.
86	Toews et al (2018)	Incarcerated women	16	Iowa, United States	Mixed Methods	Mental Health	Participants transplanted houseplants during a "planting party" which lasted 60 minutes.	Planting improved happiness, calmness, and peacefulness.
87	Odeh et al (2022)	Women	32	Florida, United States	Intervention	Mental Health	Participants took part in art activities or indoor gardening, twice a week for eight weeks.	Significant decreases in mood, perceived stress, depression, and trait anxiety after the gardening intervention.
88	Dreyer et al (2018)	Employees	214	Canada	Cross-sectional	Mental Health		Satisfaction with indoor environment in green building associated with greater hedonic wellbeing, greater eudemonic

Ref.	Author(s) (Year)	Population	N	Location	Study type	Health Outcome or Behavior	Intervention description (if applicable)	Results
								wellbeing, and decreased negative wellbeing.
90	Zijlema et al (2018)	Adults	3599	Europe	Cross-sectional	Mental Health		Commuting through a natural environment, daily associated with better mental health.
91	Chen et al (2020)	Elderly adults	349	Taipei, Taiwan	Cross-sectional	Physical Heath		Houses that did not have houseplants had significantly more particulate matter and total volatile compounds in the air than homes that had houseplants. A greater amount of particulate matter and total volatile compounds was further associated with greater heart rate and blood pressure.
92	Pedrinolla et al (2019)	Alzhiemers disease patients	163	Mantua, Italy	Intervention	Physical Heath	Participants in intervention group walked around indoor nature for five days each week for six months.	Significant decrease in salivary cortisol among the intervention group at the end of the intervention.

	Ref.	Author(s) (Year)	Population	N	Location	Study type	Health Outcome or Behavior	Intervention description (if applicable)	Results
Intentional Interactions									
	22	White et al (2021)	Adults	16,307	18 countries	Cross-sectional	Mental Health		Greater frequency of intentional interactions with nature positively associated with mental wellbeing and inversely associated with mental distress and use of medication for depression.
	23	Sidenius et al (2017)	Adults unable to work due to stress or stress-related symptoms	42	Denmark	Intervention	Physical Health	Individuals participated in nature-based therapy for 10 weeks.	Significant increase in self-reported health after the intervention.
	24	Lesser et al (2021)	Cancer survivors	114	Canada	Cross-sectional	Physical Activity and		Outdoor physical activity associated with subjective happiness and quality of life but not anxiety.

Ref.	Author(s) (Year)	Population	N	Location	Study type	Health Outcome or Behavior	Intervention description (if applicable)	Results
						Mental Health		
25	Staub et al (2019)	University students	593	United States	Longitudinal	Dietary Behavior		Gardening associated with fruit and vegetable intake.
49	Yeh et al (2018)	Older Adults	274	Taichung City, Taiwan	Cross-sectional	Physical Activity		Individuals engage in physical activity more in parks.

Ref.	Author(s) (Year)	Population	N	Location	Study type	Health Outcome or Behavior	Intervention description (if applicable)	Results
93	Maharja et al (2023)	Adults	308	Indonesi a	Cross-sectional	Mental Health		Among individuals from costal areas, swimming and snorkeling was significantly associated with mental wellbeing.
94	Marselle et al (2013)	Adults	708	England	Cross-sectional	Mental Health		Walk in green environments associated with greater mental wellbeing, less stress, and less negative affect.
95	Marselle et al (2019)	Adults	1516	United Kingdom	Cross-sectional	Physical Activity and Mental Health		Group walks in nature associated with reduced negative affect, stress, and depression and greater positive affect and mental wellbeing and reduced participants depression from recent stressful events.
96	Marselle et al (2014)	Adults	1516	England, United Kingdom	Longitudinal	Mental Health		Groups walks in nature were significantly associated with lower depression, stress, negative affect, and greater positive affect and mental-wellbeing. Frequency of walking in nature associated with lower depression, perceived stress and negative affect. Duration of walking in nature associated with greater positive affect.

Ref.	Author(s) (Year)	Population	N	Location	Study type	Health Outcome or Behavior	Intervention description (if applicable)	Results
97	Liao et al (2020)	Caregivers	42	Illinois, United States	Cross-sectional	Mental Health		Staff members reported that compared to those that did not have free garden use, dementia patients that were able to use the garden anytime had better mood, improved anger/aggression, and anxiety/agitation.
98	Wood et al (2016)	Adults	269	England, United Kingdom	Cross-sectional	Mental and Physical Health		Mood improved while gardening. Gardeners had lower weight and BMI compared to non-gardeners. Gardeners also reported better mood, lower depression, less fatigue, higher vigor and better general health than non-gardeners.
99	Schlemmer et al (2021)	Adults	349	Tyrol, Austria	Cross-sectional	Mental Health		Significant improvement in mood, calmness, and wakefulness after being in an outdoor adventure park.
100	Ku et al (2016)	Older Adults	1268	Taiwan	Longitudinal	Mental Health		Gardening associated with subjective wellbeing.
101	Fastame et al (2017)	Older Adults	406	Italy	Cross-sectional	Mental Health		Gardening associated with subjective well-being.
102	Gustafsson et al (2017)	Parents	1463	Finland	Cross-sectional	Mental Health		Significant relationship between wellbeing and visiting nature.
103	McCartan et al (2023)	Adults	1,791	United Kingdom and	Cross-sectional	Mental Health		Spending time in nature in the early morning associated with greater wellbeing.

Ref.	Author(s) (Year)	Population	N	Location	Study type	Health Outcome or Behavior	Intervention description (if applicable)	Results
				United States				
104	Li et al (2022)	Adults	133	Germany	Longitudinal	Mental Health		Being outdoors associated with greater positive affect and lower negative affect.
105	Cervinka et al (2023)	Adults	99	Australia	Cross-sectional	Mental Health		Significant increase in positive affect and mindfulness. Significant decrease in negative affect and perceived stress.
106	Edwards et al (2013)	Adults with Dementia	10	Australia	Longitudinal	Mental Health		Significant improvement in quality of life, depression, and agitation.
107	Callegari et al (2016)	Psychiatric patients	55	Lombardy, Italy	Longitudinal	Mental Health		Increase in quality of life for those that helped build a therapeutic garden.
108	Ower et al (2019)	Adults	520	Innsbruck, Austria	Cross-sectional	Mental Health		Taking part in physical activity in an alpine environment associated with quality of life.
109	Anderson et al (2018)	Students	119	California, United States	Longitudinal	Mental Health		Higher satisfaction with life was reported on days that participants interacted with nature.
110	Bu et al (2021)	Adults	55,204	United Kingdom	Longitudinal	Mental Health		Increases in gardening associated with decreases in depression and anxiety and increase in life satisfaction.
111	van den Berg et al (2016)	Adults	3748	Across Europe	Cross-sectional	Mental Health		Significant association between spending time in nature and mental health and vitality.

Ref.	Author(s) (Year)	Population	N	Location	Study type	Health Outcome or Behavior	Intervention description (if applicable)	Results
112	Phulkerd et al (2023)	Older Adults	1197	Thailand	Cross-sectional	Mental Health		Home gardening associated with happiness.
113	Machida et al (2019)	Older Adults	1000	Japan	Cross-sectional	Mental Health, Physical Activity, and Dietary Behavior		Home gardening associated with subjective happiness; greater physical activity; and eating breakfast, balanced meals, and vegetables. Community gardening associated with greater physical activity and eating enough vegetables.
114	Liu et al (2017)	Adults	308	China	Cross-sectional	Mental Health		Interacting with nature associated with relaxation.
115	Colley et al (2016)	Employees	366	Scotland, United Kingdom	Cross-sectional	Mental Health		Duration of spending time in nature associated with perceived work stress. Access to garden associated with restorativeness.
116	Ruiu et al (2022)	Older Adults	318	Italy	Cross-sectional	Physical Activity		Gardening associated with less depressive symptoms.
117	Corley et al (2021)	Adults	171	Scotland, United Kingdom	Cross-sectional	Mental Health, Physical Health, and Sleep		Those who garden were less likely to have a history of depression and cardiovascular disease but a greater body mass index. Being in the garden more often associated with greater physical health, emotional and mental health, and sleep quality.
118	Boyes (2013)	Older Adults	80	New Zealand	Mixed Methods	Mental Health and Sleep		Engagement in outdoor adventure program associated with improved sleep patterns and stress reduction.
119	Wang et al (2022)	Older Adults	12,280	China	Longitudinal	Mental Health		Gardening associated with decreased risk of cognitive impairment.

Ref.	Author(s) (Year)	Population	N	Location	Study type	Health Outcome or Behavior	Intervention description (if applicable)	Results
120	Zhang, Feng et al (2023)	Older Adults	20,912	China	Longitudinal	Mental Health		Gardening associated with reduced risk of cognitive impairment among urban residents.
121	Veldheer et al (2023)	Older Adults	146,047	United States	Cross-sectional	Mental Health, Physical Health, and Dietary Behavior		Compared to those who do not exercise, those who do garden have lower odds of cardiovascular disease, stroke, heart attack, high cholesterol, high blood pressure, diabetes, body mass index greater than or equal to 25, poor mental heath status, poor physical health status, high ten year mortality risk. In addition those that garden have greater odds of consuming fruits and vegetables five or more times per day.
122	Cox et al (2017)	Adults	1000	Southern England, UK	Cross-sectional	Mental Health		Greater frequency and duration of intentional interactions with nature inversely associated with depression.

Ref.	Author(s) (Year)	Population	N	Location	Study type	Health Outcome or Behavior	Intervention description (if applicable)	Results
123	Pearson et al (2021)	Breast Cancer Patients	56	United States	Cross-sectional	Mental Health		Decreased time in parks and trails associated with higher stress. Increased use of backyards and porches associated with lower stress. Increased use of backyard and porches associated with lower distress.
124	Roberts et al (2017)	Adults	186	United States	Longitudinal	Mental Health		Significant decrease in distress after 18 months of outdoor behavioral care.
125	Haider et al (2021)	Adults	652	Australia	Cross-sectional	Mental Health		Spending more than or equal to 60 minutes per day intentionally interacting with nature positively associated with mental wellbeing and inversely associated with experiencing depression.
126	Fjaestad et al (2023)	Mid to Older Adults	4,919	Australia	Longitudinal	Mental Health		Time spent gardening associated with mental wellbeing and life satisfaction.

Ref.	Author(s) (Year)	Population	N	Location	Study type	Health Outcome or Behavior	Intervention description (if applicable)	Results
127	Petrunoff, Yi et al (2021)	Adults	3435	Singapore	Cross-sectional	Mental Health		Duration of park use associated with wellbeing.
128	Yuen and Jenkins (2020)	Adults	94	Alabama, United States	Cross-sectional	Mental Health		Time spent in park associated with satisfaction with life and subjective wellbeing.
129	Aliyas (2021)	Older Adults	978	Iran	Cross-sectional	Mental and Physical Health		Duration of time intentionally interacting with nature associated with greater physical and mental health. Frequency of interacting with nature not associated with physical and mental health. Duration of intentional interaction with nature inversely associated with blood pressure and cardiovascular disease.
130	van den Berg et al (2019)	Adults	3948	Europe	Cross-sectional	Mental Health		Time spent in greenspace associated with mental health and vitality.
131	Ibsen et al (2021)	Individuals with dementia	94	Norway	Cross-sectional	Mental Health		Duration of time spent in nature associated with quality of life.
132	Fastame et al (2019)	Older Adults	94	Italy	Cross-sectional	Mental Health		Duration of gardening associated with decreased depression.

Ref.	Author(s) (Year)	Population	N	Location	Study type	Health Outcome or Behavior	Intervention description (if applicable)	Results
133	Shanahan et al (2016)	Adults	1538	Brisbane, Australia	Cross-sectional	Physical Health and Activity		Spending time in nature for at least 30 minutes per week could prevent 7% of cases of depression and 9% of cases of high blood pressure. Greater duration of spending time in nature associated with engagement in physical activity.
134	White et al (2019)	Adults	20,264	United Kingdom	Cross-sectional	Physical Health		Spending 120 minutes per week associated with good self-reported health.
135	Colléony et al (2020)	Adults	1023	Israel	Cross-sectional	Mental Health		Making an effort to interact with nature (e.g., taking pictures of nature) associated with positive affect.
136	Young et al (2022)	Adults	20,012	United States	Longitudinal	Mental Health		Spending less time outdoors associated with increases in depression and anxiety. Spending more time outdoors associated with increases in anxiety.
137	Saw et al (2015)	Young Adults/Students	497	Singapore	Cross-sectional	Mental Health		Use of greenspace not associated with well-being.
138	Torres et al (2016)	Adults	2,903	United States	Cross-sectional	Mental Health		Gardening not associated with depressive symptoms.

Ref.	Author(s) (Year)	Population	N	Location	Study type	Health Outcome or Behavior	Intervention description (if applicable)	Results
139	Camargo et al (2017)	Adults, Park users	1392	Colombia	Cross-sectional	Mental Health		Frequency of interacting with nature not associated with quality of life.
140	de Souto Barreto et al (2018)	Older Adults	420	France	Longitudinal	Mental Health		Gardening not associated with cognition.
141	Wang et al (2023)	Adults	500	Hangzho u, China	Cross-sectional	Mental Health		Walking outdoors inversely associated with worse mental health and use of psychotropic drugs.
142	Wei et al (2021)	Mothers	2,233	Bejing, China	Cross-sectional	Mental Health		Living close to a park or greenspace was a predictor of worse mental health.

Ref.	Author(s) (Year)	Population	N	Location	Study type	Health Outcome or Behavior	Intervention description (if applicable)	Results
143	Afrad and Kawazoe (2020)	Adults	388	Tangier, Morocco	Cross-sectional	Mental Health		Taking care of potted street gardens associated with greater depression levels.
144	Ward Thompson et al (2016)	Adults	406	Scotland, United Kingdom	Cross-sectional	Mental Health		Having a garden is a significant predictor of stress.
145	Gerber et al (2017)	Refugees	50	United States	Cross-sectional	Mental and Physical Health		No significant differences in mental and physical health between gardeners and non-gardeners.
146	Olszewska-Guizzo et al (2021)	Adults	25	Singapor e	Longitudinal	Mental Health		Individuals with high nature exposure during stay at home orders had significant decrease in Frontal Alpha Asymmetry, a brain wave indicative of positive emotions.
147	Duvall and Kaplan (2014)	Military Veterans	98	United States	Intervention	Mental Health	Participants went out into nature for 4-7 days within groups of 5-10 people.	Significant increase in positive affect, attention functioning and wellbeing and decrease in negative affect.
148	Maund et al (2019)	Adults with anxiety or depression	18	Gloucest ershire, UK	Mixed Methods	Mental Health	Six-week wetlands intervention with 2 hour sessions.	Significant increase in mental wellbeing and positive affect and decrease in negative affect and perceived stress. Participating in the intervention reduced their social isolation.

Ref.	Author(s) (Year)	Population	N	Location	Study type	Health Outcome or Behavior	Intervention description (if applicable)	Results
149	Ho et al (2022)	Adults	90	Hong Kong, China	Intervention	Mental Health	Ten-day nature-based program between noon and 2 pm.	Significant decrease in depression and anxiety and significant increase in positive affect, wellbeing, and satisfaction with life.
150	Keenan et al (2021)	Women	30	United Kingdom	Intervention	Mental Health	Participants walked in a natural or urban environment.	Significant increase in wellbeing and positive affect; significant decrease in negative affect.
151	Sahlin et al (2015)	Adults with stress related mental disorders	102	Sweden	Intervention	Mental Health	Sixteen weeks of rehabilitation in nature and 12 weeks of slowly returning to work.	Significant increase in wellbeing and decreases in depression, anxiety, and burnout.
152	Owens and Bunce (2022)	University students	68	United Kingdom	Intervention	Mental Health	Participants either completed nature-based meditation, indoor meditation or an audio tour guide (control) for 20 minutes.	Significant decreases in depression and rumination and increase in wellbeing
153	Smyth et al (2022)	Adults	897	United Kingdom	Intervention	Mental Health	Green Gym program from the United Kingdom. No control	Significant increase in wellbeing.
154	Sia et al (2020)	Older Adults	47	Singapor e	Intervention	Mental Health	24-session weekly therapy horticultural program comprising an equal mix of horticultural-based and nature art activities.	Significant increase in happiness level, mental state, cognitive function, and anxiety.
155	Down et al (2022)	University students	54	Australia	Intervention	Mental Health	Participants took part in a three day, two night expedition in nature.	Wellbeing significantly increased after the expedition.
156	Kolster et al (2023)	Adults	79	Finland	Intervention	Mental Health	Participants either took part in a nature program or a sports program.	Significant increase in mental wellbeing among individuals

Ref.	Author(s) (Year)	Population	N	Location	Study type	Health Outcome or Behavior	Intervention description (if applicable)	Results
								who took part in the nature program.
157	Stigsdotter et al (2018)	Adults	76	Denmark	Intervention	Mental Health	Participants received cognitive therapy or nature therapy intervention.	Significant improvement in both psychological wellbeing in both nature intervention and cognitive behavior therapy.
158	Yoshino et al (2018)	Adults	52	California, United States	Intervention	Mental Health	Guided walks in San Fransico.	Significant increase in positive affect and decrease in stress and cortisol.
159	Koselka et al (2019)	Adults	38	Illinois, United States	Intervention	Mental Health	Participants walked in nature for 50 minutes in the forest or on a sidewalk (intervention) and did activities of daily living in the lab (control).	Following the forest walk, increase in positive affect, and decrease in negative affect, state anxiety, and perceived stress.
160	Fuegen and Breitenbecher (2018)	Students	181	United States	Intervention	Mental Health	Participants walked or rested indoors or outdoors.	Exercising and resting in nature increased positive affect.

Ref.	Author(s) (Year)	Population	N	Location	Study type	Health Outcome or Behavior	Intervention description (if applicable)	Results
161	Reeves et al (2019)	Adults	34	London, United Kingdom	Intervention	Mental and Physical Health	Participants experienced wetlands, indoors or were in control group for 20 minutes for six weeks.	Significant increase in positive affect and decrease in negative feelings after wetlands intervention. No significant differences in physiological measurements between groups.
162	Li et al (2021)	Adults	48	China	Intervention	Mental and Physical Health	Participants walked in a green or urban environment.	Significant increase in positive affect, vigor, and restorativeness in those that walked in green environment. Significant decrease in systolic and diastolic blood pressure, and mean arterial pressure.
163	Song et al (2018)	Adults	585	Japan	Intervention	Mental Health	Participants walked in nature or urban area for 15 minutes.	When compared to walking in an urban area, walking in the forest has been associated with decreased depression, anxiety, anger-hostility, fatigue, confusion, and greater vigor.
164	Schramm et al (2022)	Adults with major depressive disorder	61	Germany	Intervention	Mental Health	Nature and animal assisted mindfulness program or treatment as usual for one year.	Participants in intervention experienced decreased depression and greater quality of life. Additionally, they experienced decreased depression relapses and fewer weeks with major depressive disorder when compared to those who received usual care
165	Wheeler et al (2020)	Military Veterans	18	United Kingdom	Intervention	Mental Health	Participants in intervention group went angling.	Lower post traumatic stress disorder symptoms, depression, anxiety, and perceived stress after intervention.

Ref.	Author(s) (Year)	Population	N	Location	Study type	Health Outcome or Behavior	Intervention description (if applicable)	Results
166	Rosenberg et al (2014)	Cancer Survivors	199	United States	Intervention	Mental and Physical Health	Participants took part in a six day outdoor adventure program.	Significant improvement in body image, self-compassion, self-esteem, discomfort, depression, fatigue, memory, and somatic anxiety.
167	Grafetstätter et al (2017)	Health care professionals	91	Austria	Intervention	Mental Health	Participants of the intervention groups hiked in nature for 6 days for one hour.	Significant improvement in aggression, obsession-compulsive, depression, paranoid ideation, phobic anxiety, and interpersonal sensitivity.
168	Lacharité-Lemieux et al (2015)	Postmenopausal women	23	Canada	Intervention	Mental Health and Physical Activity	Participants exercised outdoors or indoors, three times a week for 12 weeks.	Depression scores significantly decreased and physical activity significantly increased among those who exercised outdoors.
169	Bird (2015)	Veterans	20	Australia	Intervention	Mental Health	Participants spent five days in nature.	Significant decrease in depression, anxiety and stress after the intervention.
170	Song et al (2019)	Female students	72	Japan	Intervention	Physical Health	Participants walked in a natural or urban environment for 15 minutes.	Individuals who walked in natural environment experienced greater parasympathetic nervous activity and decreased sympathetic nervous activity and heart rate.

Ref.	Author(s) (Year)	Population	N	Location	Study type	Health Outcome or Behavior	Intervention description (if applicable)	Results
171	Lee et al (2014)	Men	48	Japan	Intervention	Physical Health	Participants walked in nature for 12-15 minutes over two days.	Significant increase in parasympathetic nervous activity. Significant decreases in sympathetic nervous activity, heart rate, systolic blood pressure, and anxiety.
172	Song, Ikei, Igarashi et al (2015)	Adults	23	Japan	Intervention	Mental and Physical Health	One group walked in an urban park area and then throughout the city. Another group walked through the city then the urban park.	Participants felt significantly more relaxed and vigor, and less anxiety, anger, fatigue, and confusion after walking in the park compared to urban area. Significantly greater parasympathetic nervous activity and lower sympathetic nervous activity and heart rate while walking in park area compared to when walking in the urban area.
173	Song et al (2013)	Undergraduate Students	13	Japan	Intervention	Mental Health	Participants walked in urban parks and city areas	Significantly greater parasympathetic nervous activity when walking in park. Significantly less anxiety and more relaxed after walking in park.
174	Kotozaki (2020)	Postpartum women	15	Japan	Intervention	Mental Health	Participant took part in gardening activities for eight weeks.	Significant decrease in state and trait anxiety, total mood disturbance, and postpartum depression.
175	Vujcic et al (2017)	Psychiatric patients	30	Belgrade, Serbia	Intervention	Mental Health	Nature based therapy program for one hour, three days a week, for three weeks.	Significant decrease in stress.

Ref.	Author(s) (Year)	Population	N	Location	Study type	Health Outcome or Behavior	Intervention description (if applicable)	Results
176	Høegmark et al (2021)	Men with deleterious mental health or long-term illness	153	Denmark	Intervention	Mental Health	Nine-week nature intervention. Participants met once a week for three hours. Control group received treatment as usual	Significant decrease in stress and increases in physical and psychological quality of life and restorativeness.
177	Razani et al (2018)	Parents	78	United States	Intervention	Mental Health	Both sets of participants received a park prescription. One group "filled" their prescription with a group.	Significant decrease in stress, loneliness, and cortisol. Significant increase in moderate to vigorous physical activity.
178	Anzman-Frasca et al (2023)	Adults	104	New York, United States	Intervention	Mental Health and Sleep	Intervention group received access to a winter hiking challenge for 12 weeks.	Greater frequency of hiking associated with more hours of sleep and less stress.
179	Hassan and Deshun (2023)	Students	50	Shanghai, China	Intervention	Mental and Physical Health	Participants completed a mental task or watered plants.	After watering plants, participants experienced a significant decrease in systolic and diastolic blood pressure; and experienced tranquility, focus, less stress, and greater relaxation.
180	Vella et al (2013)	Veterans with Post Traumatic Stress Disorder	74	Utah, United States	Intervention	Mental Health and Sleep	Participants went fly fishing for two days and three nights.	Significant improvements in psychological distress, mood, perceived stress, and sleep quality.
181	Mutz and Muller (2016)	Students	15	Germany	Intervention	Mental Health	Participants went on a seven day nature excursion.	Significant improvement in stress, life satisfaction, and happiness.

Ref.	Author(s) (Year)	Population	N	Location	Study type	Health Outcome or Behavior	Intervention description (if applicable)	Results
182	Yang and Conroy (2019)	Older Adults	29	United States	Intervention	Mental Health	Participants walked in nature for 30 minutes, eight times.	Significant reduction in negative affect.
183	Watkins-Martin et al (2022)	Adults with Major Depressive Disorder	37	Quebec, Canada	Intervention	Mental Health	Participants walked in nature or urban area for 60 minutes.	Significant decrease in negative affect after walking in nature condition.
184	Coss and Keller (2022)	Adults	105	California, United States	Intervention	Mental and Physical Health	Participants viewed bodies of water or other landmarks.	Viewing water is associated with decreased blood pressure, decreased heart rate, and increased relaxation.
185	Pfeifer et al (2019)	Students	84	Freiburg, Germany	Intervention	Mental Health	Participants had music therapy and seminars in nature.	Significant increase in relaxation.
186	Pfeifer et al (2020)	Students	46	Freiburg, Germany	Intervention	Mental Health	Participants silently sat in nature or indoors.	Significant increase in relaxation while in nature.
187	Olszewska-Guizzo et al (2022)	Adults	74	Singapore	Intervention	Mental Health	Participants were exposed to nature indoors and in a lab.	Brain activation (mindfulness, relaxation, and attention restoration) associated with the therapeutic value of landscapes in an outdoor environment.
188	McCaffrey and Liehr (2016)	Adults with psychological stress	195	Florida, United States	Intervention	Mental Health	Six week garden walking program.	Significant increase in hope, personal growth and quality of life.
189	Petrunnoff, Yao et al (2021)	Adults	160	Singapore	Intervention	Mental Health	Participants in the intervention were given a park exercise prescription.	Park physical activity mediated the effect of the park prescription program on psychological quality of life.

Ref.	Author(s) (Year)	Population	N	Location	Study type	Health Outcome or Behavior	Intervention description (if applicable)	Results
190	Flowers et al (2016)	Adults	2079	United Kingdom	Cross-sectional	Physical Activity		Greater frequency of intentional interactions has been associated with meeting United Kingdom physical activity guidelines.
191	Ojala et al (2019)	Adults	88	Helsinki, Finland	Intervention	Mental Health	Participants were exposed to three different settings: urban area, urban park and woodland.	The forest and park were perceived as more restorative and having greater vitality than the urban environment.
192	Brown et al (2020)	Native American adutls	32	Montana, United States	Intervention	Mental Health	A garden was installed and 10 sessions taught participants about gardening and food.	Change in mood disturbance was significantly different between intervention and control group; such that intervention group had a decrease in mood disturbance and control had an increase in mood disturbance.
193	Djernis et al (2021)	University students	60	Denmark	Intervention	Mental Health	Five day residential program where participants received the program in nature or indoors. Control group did not receive any intervention.	Significant improvement in self-compassion.
194	Hewitt et al (2013)	Adults with Young-Onset Dementia	12	United Kingdom	Intervention	Mental Health	Participants took part on a year long gardening program.	Significant improvement in mini mental state exam.

Ref.	Author(s) (Year)	Population	N	Location	Study type	Health Outcome or Behavior	Intervention description (if applicable)	Results
195	Park et al (2020)	Older Adults	40	Seoul, South Korea	Intervention	Physical Health	Participants in the intervention group completed a 24-session gardening program.	Significant increase in brain-derived neurotrophic factor, improvement in hand dexterity, and decrease in task time among the intervention group. Significantly greater Korean Mini Mental State Examination score in the intervention group compared to the control group.
196	Hyvonen et al (2023)	Adults	134	Finland	Intervention	Mental Health	Participants in the intervention had 12 nature-based sessions. Control group received standard care.	Psychological distress significantly decreased among intervention group.
197	Aspinall et al (2015)	Adults	12	United Kingdom	Intervention	Mental Health	Participants walked in urban shopping street, green space, then busy commercial street.	Reduction in arousal, frustration, and directed attention and increase in meditation when transitioning from urban street to green space.
198	Neale et al (2017)	Older Adults	95	Edinburg h, Scotland	Intervention	Mental Health	Participants walked in urban and natural environments.	There were higher levels of engagement when walking in natural environment compared to walking in urban environments.
199	Piñeyro Salvidegoitia et al (2019)	Adults	25	Germany	Intervention	Mental Health	Participants walked indoors and outdoors.	Being in a natural environment improves recall.
200	Lin et al (2020)	Students	40	China	Intervention	Mental Health	After doing a learning task, participants walked or sat in nature.	Participants of the walking group had greater valence and meditation.
201	Lymeus et al (2018)	Adults	80	Sweden	Intervention	Mental Health	Participants meditated in an indoor or nature environment.	Improved attention performance after meditating in nature.

Ref.	Author(s) (Year)	Population	N	Location	Study type	Health Outcome or Behavior	Intervention description (if applicable)	Results
202	Boere et al (2023)	Students	30	Victoria, British Columbia, Canada	Intervention	Mental Health	Participants walked outdoors or indoors.	Significant increase in working memory and attention.
203	LoTemplio et al (2020)	Adults	73	United States	Intervention	Mental Health	Participants took part in five day camping trip.	Significant increase in error-related negativity which has been associated with higher working memory capacity and cognitive ability.
204	Gagliardi et al (2019)	Older Adults	112	Italy	Intervention	Mental Health	Participants took part in social farming for one year.	No change in quality of life.
205	Largo-Wight et al (2017)	Employees	37	United States	Intervention	Mental Health	Intervention group were prompted to take a ten minute break each day and be aware of the nature around them. Control group took a ten minute break indoors.	No difference in stress for intervention and control group after the breaks.
206	Willert et al (2014)	Adults on long-term sick leave	93	Denmark	Intervention	Mental Health	Participants took part in a job rehabilitation center or garden program.	No significant differences in mental health outcomes between the groups.

Ref.	Author(s) (Year)	Population	N	Location	Study type	Health Outcome or Behavior	Intervention description (if applicable)	Results
207	Smiley et al (2020)	Adults	1527	Indiana, United States	Cross-sectional	Physical Health, Physical Activity, and Sleep		Trail usage was significantly related with more physical activity, better sleep quality, and stronger self-related overall wellness and health.
208	Adjei and Brand (2018)	Adults	36,240	25 countries	Cross-sectional	Physical Health		Individuals who spend time gardening report greater self-reported health.
209	Pitas et al (2017)	Adults	1144	United States	Cross-sectional	Physical Health		Frequent park use associated with high self-rated reported health.
210	Dzhambov et al (2023)	Adults	917	Bulgaria	Cross-sectional	Physical Health		Greater duration of intentional interactions with nature are associated with decreased negative self reported health.
211	Ogura et al (2022)	Older Adults	41	Japan	Longitudinal	Physical Health		Gardeners were able to maintain their physical function despite disruption in cardiac rehabilitation.
212	Felson et al (2021)	Adults	1304	United States	Longitudinal	Physical Health		Resolved frequent knee pain associated with gardening.
213	Gaskins et al (2014)	Males	231	Massachusetts, United States	Cross-sectional	Physical Health		Greater duration of time in outdoor activities associated with greater sperm concentration.
214	Lêng and Wang (2013)	Older Adults	5464	Taiwan	Longitudinal	Physical Health		Gardening associated with mobility.
215	Dzhambov, Markevych & Lercher (2018)	Adults	555	Australia	Cross-sectional	Physical Health		Having a garden associated with lower diastolic blood pressure.

Ref.	Author(s) (Year)	Population	N	Location	Study type	Health Outcome or Behavior	Intervention description (if applicable)	Results
216	Kingsley et al (2022)	Adults	4,614	Australia	Cross-sectional	Physical Health		Compared to those who do not garden, individuals who garden had less cardiometabolic risk, lower waist circumference, diastolic blood pressure, and triglycerides.
217	Kegler et al (2020)	Adults	3889	United States	Cross-sectional	Physical Health and Dietary Behavior		Gardening associated with fruit and vegetable intake and lower body mass index.
218	Zick et al (2013)	Adults	198	Utah, United States	Cross-sectional	Physical Health		Gardeners had significantly lower body mass index than their non-gardening neighbors.
219	Hofmann et al (2018)	Gardeners	85	Switzerla nd	Longitudinal	Physcial Health and Activity		Significant relationships between duration of interacting with nature, engagement in physical activity and decrease in cortisol.
220	Zhao et al (2023)	Older Adults	353	Jinan, China	Cross-sectional	Physical Health		Women with anxiety were less likely to develop frailty when their nursing home provided outdoor activity spaces.
221	Armstrong et al (2020)	Postmenopausal women	371,27 9	United Kingdom	Cross-sectional	Physical Health		Gardening associated with less risk of lower limb and hip fracture.
222	Føns Johnsen et al (2013)	Older Adults	55,705	Denmark	Longitudinal	Physical Health		Gardening associated with lower mortality.
223	Lee (2013)	Older Adults	624	United States	Longitudinal	Physical Health		Gardening often associated with a reduced risk of mortality.
224	Ratjen et al (2017)	Cancer Survivors	1376	Germany	Longitudinal	Physical Health		Gardening inversely associated with all-cause mortality.

Ref.	Author(s) (Year)	Population	N	Location	Study type	Health Outcome or Behavior	Intervention description (if applicable)	Results
225	Ahmadi-Abhari et al(2017)	Adults	5196	United Kingdom	Longitudinal	Physical Health		Gardening not associated with change in arterial stiffness over 5 years.
226	Noushad et al (2022)	Adults	262	Pakistan	Intervention	Physical Health	Participants walked or sat in nature for one hour for five days over three months.	Significant decreases in cortisol, c reactive protein, and interleukin 6.
227	Coolman et al (2020)	Adults	186	Missouri, United States	Intervention	Physical Health	Participants walked through a zoo trail.	Significant decrease in cortisol, systolic and diastolic blood pressure.
228	Han et al (2018)	Older adults with mental health	28	South Korea	Intervention	Physical Health	Participants in intervention group took part on 10-week horticultural therapy program.	Significant decrease in cortisol among the intervention group. Significant increase in Senior Fitness Test among the intervention group. No significant changes in control group.
229	Hunter et al (2019)	Adults	36	Michigan, United States	Intervention	Physical Health	Participants were asked to spend at least 10 minutes in nature for 3 days a week, over an 8-week period.	Intentionally interacting with nature for at least 20-30 minutes results in an 18.5% decrease in cortisol per hour.
230	Lipponen et al (2022)	Female health care workers	11	Finland	Intervention	Physical Health	Six nature-based interventions occurred over a six-month period. No control group.	Significant decrease in cortisol on intervention days but not over the course of the intervention period. Significant increase in salivary alpha amylase and no change in heart rate variability.

Ref.	Author(s) (Year)	Population	N	Location	Study type	Health Outcome or Behavior	Intervention description (if applicable)	Results
231	Beil and Hanes (2013)	Adults	15	Oregon, United States	Intervention	Physical Health	Participants sat in one of four environments ranging from very built to very natural for 20 minutes.	Significantly greater alpha amylase concentration among individuals who sat in very built environment. No difference in cortisol concentrations between sitting in the four environments.
232	Song et al (2015)	Men	20	Japan	Intervention	Physical Health	Participants walked in a natural or urban environment over 17 minutes.	Individuals who walked in the forest environment experienced greater parasympathetic activity and decreased heart rate.
233	de Brito et al (2020)	Adults	24	Minnesota, United States	Intervention	Physical Health	Participants walked in nature and in an urban area.	Walking in nature increased heart rate variability.
234	Grazuleviciene et al (2015)	Coronary Artery Disease patients	20	Lithuania	Intervention	Physical Health	Urban exposure group walked along a busy street. Green exposure group walked through a park. Both groups walked for 30 minutes for 7 days.	Significant decrease in systolic and diastolic blood pressure among green exposure group. Significant increase in heart rate recovery in the green exposure group.
235	Lidén et al (2016)	Women	52	Sweden	Intervention	Mental and Physical Health	Participants took part in horticulture therapy and supported employment.	Significant improvement in pain, general health, vitality, and mental health.
236	Suenaga et al (2020)	Students	38	Japan	Intervention	Physical Health	Participants walked in nature.	Significant decrease in sympathetic nervous activity.

Ref.	Author(s) (Year)	Population	N	Location	Study type	Health Outcome or Behavior	Intervention description (if applicable)	Results
237	Tsao et al (2022)	Adults	25	Taiwan	Intervention	Physical Health	Participants took part in forest bathing or walking in an urban park.	Among those who participated in forest bathing, there was a significant decrease in systolic blood pressure, central end systolic blood pressure, pulse pressure, heart rate, cardiac function, and vascular function. No significant change in cardiovascular function after walking in an urban park. Heart rate was significantly lower among those that walked in the forest compared to the urban park.
238	Lanki et al (2017)	Women	36	Finland	Intervention	Physical Health	Participants visited an urban forest, urban park, or city center for 45 minutes.	Sitting in the forest was associated with lower systolic blood pressure. Visiting the greenspaces was associated with lower heart rate.
239	Navalta et al (2021)	Students	10	Nevada, United States	Intervention	Physical Health	Participants walked in one of five environments for 30 minutes.	Significantly lower heart rate when walking in green environment when compared to urban environment. Significantly greater comfort reported after walking in brown environment compared to green.
240	Sahlin et al (2014)	Women	33	Sweden	Mixed Methods	Physical Health	Nature-based stress management course that met for 3 hours, twice a week over 12 weeks.	Significant decrease in gastrointestinal symptoms.

Ref.	Author(s) (Year)	Population	N	Location	Study type	Health Outcome or Behavior	Intervention description (if applicable)	Results
241	Park et al (2019)	Older Adults	41	Seoul, South Korea	Intervention	Physical Health	Participants completed a 20 minute gardening activity.	Significant increase in brain-derived neurotrophic factor and platelet-derived growth-factor, which are related to memory.
242	Sudimac et al (2022)	Adults	63	Berlin, Germany	Intervention	Mental Health	Participants were faced with several stressful tasks while ongoing functional magnetic resonance imaging scanning. Participants then walked in a natural or urban environment and then underwent another functional magnetic resonance imaging scan while being faced with the same stressful tasks.	Individuals who had walked in a natural environment had significantly less amygdala activity during the second functional magnetic resonance imaging scan and stressful tests.
243	Burrows et al (2018)	Adults	865	Dublin, Ireland	Cross-sectional	Physical Activity		Visiting a park more often associated with engagement in physical activity at the park.
244	de Boer et al (2017)	Older Adults	115	Netherla nds	Longitudinal	Physical Activity		Residents of green care farms were more physically active than residents of traditional nursing homes.
245	Byrka and Ryczko (2018)	Adults	64	Poland	Intervention	Physical Activity	Participants salsa danced in a park or indoor environment.	Individuals who danced in the park engaged in more vigorous activity than those who danced indoors.

Ref.	Author(s) (Year)	Population	N	Location	Study type	Health Outcome or Behavior	Intervention description (if applicable)	Results
246	Boyle et al (2020)	Adults	59	Rhode Island, United States	Intervention	Physical Activity	Participants received marketing materials to promote physical activity and were instructed to walk for 30-60 minutes/day.	Greater time spent walking outdoors compared to indoors. Affective response did not mediate the relationship between walking outdoors and minutes walking.
247	Hooper et al (2020)	Mid to Older Adults	11,036	Australia	Cross-sectional	Physical Activity		Park users were more likely to meet physical activity guidelines.
248	Park et al (2017)	Adults	524	Missouri, United States	Cross-sectional	Physical Activity		Use of trails significantly associated meeting physical activity recommendations.
249	Teixeira et al (2022)	Adults	194	Portugal	Cross-sectional	Physical Activity		Greater frequency on intentionally interacting with nature associated with more steps taken per day.
250	Hughley et al (2021)	Adults	360	United States	Cross-sectional	Physical Activity		Frequency of park visits associated with engagement in moderate to vigorous physical activity.
251	Murray et al (2017)	Women	377	United States	Cross-sectional	Physical Activity and Sleep		Greater duration of intentional interactions with nature is associated with engagement in moderate to vigorous physical activity and time asleep.

Ref.	Author(s) (Year)	Population	N	Location	Study type	Health Outcome or Behavior	Intervention description (if applicable)	Results
252	Stewart et al (2016)	Adults	671	United States	Cross-sectional	Physical Activity		Park visitors had greater engagement in moderate to vigorous physical activity than non-park visitors.
253	Gill et al (2016)	Cancer survivors	82	United States	Intervention	Physical Activity	Week long outdoor adventure therapy.	Significant increase in physical activity after the outdoor adventure therapy. Significantly greater physical activity 3 months after intervention compared to the control.
254	Albujulaya and Stevinson (2023)	Adults	1046	Saudi Arabia	Cross-sectional	Physical Activity and Health		Exercising outdoors was significantly correlated with well-being.
255	Pasanen et al (2014)	Adults	2070	Finland	Cross-sectional	Mental Health, Physical Health, and Sleep		Physical activity in nature associated with emotional wellbeing, general health, and sleep quality.
256	Lawton et al (2017)	Adults	262	United Kingdom	Cross-sectional	Physical Activity and Mental Health		Exercising outdoors associated with wellbeing and lower levels of somatic anxiety.
257	Loureiro and Veloso (2014)	Adults	282	Lisbon, Portugal	Cross-sectional	Physical Activity		Individuals who exercise outdoors reported greater wellbeing and positive affect.

Ref.	Author(s) (Year)	Population	N	Location	Study type	Health Outcome or Behavior	Intervention description (if applicable)	Results
258	Puett et al (2014)	Adults	11,649	Texas, United States	Cross-sectional	Physcial Activity, Mental and Physical Health		For those that exercise, being physically active outdoors is protective of tension, worse stress management, emotional outlook, and health.
259	Holt et al (2019)	Undergraduate students	207	United States	Cross-sectional	Mental Health		Individuals who exercise outdoors report experiencing less stress, being very happy, and greater quality of life.
260	Matias et al (2022)	Adults	88,522	Brazil	Cross-sectional	Mental Health		Exercising outdoors associated with lower odds of depression.
261	Das and Gailey (2022)	Adults	8,253	United States	Longitudinal	Physical Activity and Mental Health		Exercising in a green space associated with a decrease in depression and anxiety. Whereas, exercising indoors is not associated with improved mental health.
262	Badon et al (2022)	Pregnant Women	11,033	California, United States	Cross-sectional	Physical Activity and Mental Health		Outdoor physical activity associated with decreased prevalence of depression and anxiety.
263	Lesser and Nienhuis (2020)	Adults	1098	Canada	Cross-sectional	Mental Health		Individuals who exercised outdoors during the COVID-19 pandemic had lower anxiety than those who did not exercise outdoors.

Ref.	Author(s) (Year)	Population	N	Location	Study type	Health Outcome or Behavior	Intervention description (if applicable)	Results
264	Marmett et al (2022)	Men	120	Brazil	Cross-sectional	Physical Health		Individuals who exercised outdoors had a lower heart rate and lower triglycerides than those who exercised indoors.
266	Van den Heuvel et al (2013)	Older Adults	1509	Netherla nds	Cross-sectional	Physical Health		Duration of outdoor exercise not associated with vitamin D status.
267	Calogiuri et al (2016)	Employees	14	Norway	Intervention	Physical Activity, Mental and Physical Health	Participants took part in two exercise sessions. The intervention group's session took part outside; and the control group's sessions took place indoors.	Greater positive affect and reduced cortisol and diastolic blood pressure measured among outdoor exercise group compared to the indoor exercise group.
268	Calogiuri et al (2015)	Employees	14	Norway	Mixed Methods	Physical Activity and Mental Health	Participants took part in two excercise sessions. The intervention group's session took part outside; and the control	Greater positive affect in the nature group.

Ref.	Author(s) (Year)	Population	N	Location	Study type	Health Outcome or Behavior	Intervention description (if applicable)	Results
							group's sessions took place indoors.	
269	Müller-Riemenschneider et al (2020)	Adults	160	Singapore	Intervention	Mental Health	Park prescription intervention where participants exercise outdoors and a control group.	Significant improvement in psychological quality of life.
270	Legrand et al (2018)	Individuals with depression	18	Reims, France	Intervention	Physical Activity and Mental Health	Participants exercised in an indoor or nature condition for 20 minutes or were sedentary.	No difference in energy or fatigue between those that exercised indoors or outdoors.
271	Turner and Stevinson (2017)	Adults	22	United Kingdom	Intervention	Mental Health	Participants ran inside and in a greenspace.	No significant difference in psychological effects after running in indoor or outdoor environment.
272	Klaperski et al (2019)	Student athletes	140	Germany	Cross-sectional	Mental Health		No significant differences in stress and wellbeing between indoor and outdoor exercise.
273	Colley et al (2020)	Adults	4524	Canada	Cross-sectional	Mental and Physical Health		Individuals who exercised outdoors reported better mental and physical health.
274	Folayan et al (2022)	Adults	4471	Nigeria	Cross-sectional	Physical Activity and Mental Health		Individuals who exercised outdoors during the COVID-19 pandemic had higher odds of feeling lonely.

Ref.	Author(s) (Year)	Population	N	Location	Study type	Health Outcome or Behavior	Intervention description (if applicable)	Results
275	Jenkins et al (2022)	Adults	745	New Zealand	Cross-sectional	Physical Activity and Mental Health		Exercising outdoors has no greater effect on mental wellbeing than exercising indoors.
276	Gladwell et al (2016)	Adults	13	United Kingdom	Intervention	Sleep	Participants walking in nature or in a built environment on their lunch break.	Walking in nature improved sleep quality and greater sleep restoration that night.
277	Nova et al (2020)	Adults	102	Porto, Portugal	Cross-sectional	Dietary Behavior		Gardening associated with fruit and vegetable intake.
278	Barnidge et al (2013)	Adults	1141	Missouri, United States	Cross-sectional	Dietary Behavior		Significant relationship gardening and consuming fruits and vegetables.
279	Drisdelle et al (2020)	Adults	417	Quebec, Canada	Cross-sectional	Dietary Behavior		Gardening associated with fruit and vegetable intake.
280	Litt et al (2015)	Adults	469	Colorado, United States	Cross-sectional	Dietary Behavior		Gardening associated with greater frequency of fruit and vegetable consumption.
281	Ornelas et al (2018)	Adults	169	United States	Cross-sectional	Dietary Behavior		Individuals who garden more than four times per month reported more fruit and vegetable consumption.
282	Loso et al (2018)	University students	1121	United States	Cross-sectional	Dietary Behavior		Students who garden more than once a week consumed more fruits and vegetables.

Ref.	Author(s) (Year)	Population	N	Location	Study type	Health Outcome or Behavior	Intervention description (if applicable)	Results
283	Rammohan et al (2019)	Adults	3230 house holds	Myanmar	Cross-sectional	Dietary Behavior		Having a home garden associated with greater dietary diversity.
284	Demark-Wahnefried et al (2018)	Older adult cancer survivors	46	Alabama, United States	Intervention	Dietary Behavior	Gardening intervention for one year. Waitlist control.	Individuals who received the intervention increased fruit and vegetable consumption.
285	Bail et al (2018)	Breast Cancer Survivors	82	Alabama, United States	Intervention	Dietary Behavior	Participants in intervention group were paired with a master gardener and maintained a garden for one year.	Trend toward significant increase in vegetable consumption.
286	Spees et al (2016)	Cancer Survivors	22	Ohio, United States	Intervention	Dietary Behavior	Participants took part in a four month gardening intervention.	Significant increase in fruit and vegetable consumption.
287	Alemu et al (2019)	Adults	884	Ethiopia	Intervention	Dietary Behavior	Participants in the experimental group implemental a permagarden for one year.	Greater portion of intervention participants consumed fruits and vegetables at least twice a week than control group.
288	Blair et al (2013)	Cancer survivors	112	Alabama, United States	Intervention	Dietary Behavior	Yearlong gardening intervention with a master gardener.	60% of intervention group increased their fruit and vegetable consumption by 1 cup per day.

	Ref.	Author(s) (Year)	Population	N	Location	Study type	Health Outcome or Behavior	Intervention description (if applicable)	Results
	289	Blair et al (2021)	Cancer survivors	30	Alabama, United States	Intervention	Dietary Behavior	Participants received nine months of mentoring from master-gardeners.	Increase in fruit and vegetable consumption (1.2 cup).
	290	Tharrey et al (2020)	Adults	122	Montpellier, France	Intervention	Dietary Behavior	Participants in intervention group started gardening in a community garden over one growing season.	No significant changes in fruit and vegetable consumption.
Indirect and Incidental	31	Bjørnstad et al (2016)	Employees	565	Norway	Cross-sectional	Mental Health		Significant negative relationships between indoor contact with nature (indirect and incidental interactions with nature), job stress and subjective health complaints.
	38	Garrido-Cumbrera et al (2022)	Adults	3109	Europe	Cross-sectional	Mental Health		Poorer wellbeing associated with not having nature in the home and not having views of nature.
Indirect and Intentional	35	Martin et al (2019)	Adults	149	England, United Kingdom	Cross-sectional	Dietary Behavior		Significant inverse associations between having a greenspace view, craving frequency, craving strength, and negative affect. Likewise significant associations between access to garden, craving frequency, craving strength, and negative affect.
	52	Brooks et al (2017)	Adults	284	Canada	Intervention	Mental Health	Participants walked in nature, looked at pictures, of nature, or both.	Intentionally and indirectly interacting with nature improves mood.

	Ref.	Author(s) (Year)	Population	N	Location	Study type	Health Outcome or Behavior	Intervention description (if applicable)	Results
	55	Horan et al (2023)	Employees	25	United States	Intervention	Mental Health	Participants did a task, took a break in virtual or real nature or they sat quietly (control), then did another task.	Participants who interacted with nature (virtually or actually) reported greater positive affect.
	56	Browning et al (2020)	Students	82	United States	Intervention	Mental Health	Participants either sat in nature, sat indoors while experiencing nature via virtual reality, or sat indoors with no exposure to nature.	Significant increase in positive affect and restorativeness for those that experienced nature outdoors and via virtual reality.
	79	Mavrantza et al (2023)	Adults	30	Florida, United States	Intervention	Physical Health	Participants exercised in virtual, green, or indoor space.	Greater parasympathetic activity when exercising in virtual space compared to green space.
	81	Ahnesjö et al (2022)	Older adults	48	Sweden	Intervention	Physical Health	Participants biked in three different conditions: indoors, simulated outdoor environment, and outdoors	No significant differences in heart rate between three environments.
Incidental and Intentional	89	Heilmayr and Friedman (2020)	Students	138	California, United States	Intervention	Mental and Physical Health	Participants were randomized into one of five groups and did the activity for four weeks: gardening, physical activity, being in nature, watching a film, or indoor container gardening.	While emotional wellbeing and self-reported health improved, there was no significant differences between groups.
All types	21	Cox, Hudson et al (2017)	Adults	1023	England, UK	Cross-sectional	Physical Health		All types of interactions with nature were associated with greater self-reported health.
